# Manipulation of Interleukin-6 (IL-6) and Transforming Growth Factor Beta-1(TGFβ-1) towards viral induced liver cancer pathogenesis

**DOI:** 10.1371/journal.pone.0275834

**Published:** 2022-10-10

**Authors:** Yasmin Badshah, Maria Shabbir, Khushbukhat Khan, Maha Fatima, Iqra Majoka, Laiba Aslam, Huda Munawar

**Affiliations:** Department of Healthcare Biotechnology, Atta-ur-Rahman School of Applied Biosciences, National University of Sciences and Technology, Islamabad, Pakistan; The University of Texas Rio Grande Valley, UNITED STATES

## Abstract

Hepatocellular carcinoma (HCC) is the most common liver malignancy. Early diagnosis of HCC has always been challenging. This study aims to assess the pathogenicity and the prevalence of IL-6 -174G/C (rs1800795) and TGFβ-1 +29C/T (rs1800470) polymorphisms in HCV-infected HCC patients. Experimental strategies are integrated with computational approaches to analyse the pathogenicity of the TGFβ-1 +29C/T and IL-6–174 G/C polymorphisms in HCV-induced HCC. AliBaba2 was used to predict the effect of IL-6–174 G/C on transcription factor binding site in IL-6 gene. Structural changes in the mutant TGFβ-1 structure were determined through project HOPE. To assess the polymorphic prevalence of IL-6 -174G/C and TGFβ-1 +29C/T genotypes in HCC and control subjects, amplification refractory mutation system PCR (ARMS-PCR) was performed on 213 HCC and 216 control samples. GraphPad Prism version 8.0 was used for the statistical analysis of the results. In-silico analysis revealed the regulatory nature of both IL-6 -174G/C and TGFβ-1 +29C/T polymorphisms. ARMS-PCR results revealed that the individuals carrying TT genotype for TGFβ-1 gene have an increased risk of developing HCC (p<0.0001, OR = 5.403, RR = 2.062) as compared to individuals with CT and CC genotype. Similarly, GC genotype carriers for IL-6 gene exhibit an increased risk of HCC susceptibility (p<0.0001, OR = 2.276, RR = 1.512) as compared to the people carrying the GG genotype. Genotype TT of TGFβ-1 gene and genotype GC of IL-6 gene are found to be associated with HCV-induced HCC. IL-6 polymorphism may alter its transcription that leads to its pathogenicity. TGFβ-1 polymorphism may alter protein structure stability.

## Introduction

Hepatocellular Carcinoma (HCC) is a form of primary liver cancer originating from hepatocytes. It is the sixth most common cancer in the world, reporting 800,000 cases annually with an average mortality rate of 700,000 people per year [1]. HCC is attributed to several causative factors including Hepatitis B and C viral infections, alcoholic liver disease, aflatoxin, autoimmune disorders, Wilson’s disease, type 2-diabetes, hemochromatosis, obesity, and previously occurring liver cirrhosis [2–5]. Hepatitis C virus (HCV) and aflatoxin are the prevalent risk factors in Asia and Africa with 70% reported cases [6, 7].

HCV is accredited to 25% of annually reported HCC cases [8]. 71 million people get infected with HCV annually, out of which 20–30% develop cirrhosis and 1–7% population ends up developing HCC [9]. Prior to its development into HCC, the HCV infection passes through a number of stages such as chronic inflammation, fibrosis, genomic alterations, and formation of tumor microenvironment [10]. The non-structural proteins of HCV upregulate tumor growth factor ß-1 (TGF-β1) signalling by activating reactive oxygen species (ROS), p38, and MAPK, JNK, ERK, & NF-κB pathways [11–13]. HCV also suppresses anti-tumor genes such as TP53, TP73, Retinoblastoma1, p53, and CDKN1A (a down-regulator of cell cycle) to develop carcinogenesis [11, 14, 15]. Although, TGFβ-1 acts as a negative growth regulator in normal cellular mechanisms [16]. It also acts as tumor-suppressor in pre-malignant cells; but it promotes cancer metastasis in advanced stage tumors. 14-3-3ζ protein has been demonstrated to inhibit the tumor suppression ability of TGFβ-1 by destabilizing the expression of p53 [17]. Polymorphisms in the TGFβ-1 gene have been studied for their association with the aetiology of multiple diseases. Studies have identified the possible role of TGFβ-1+29C/T polymorphism in the pathogenesis of osteoarthritis [18], breast cancer [19], urinary bladder cancer [20], development of cervical lesions [21], sarcopenia [22], and chronic periodontitis [23]. However, the association of the TGFβ-1+29C/T polymorphism with the development of HCV-induced HCC has not been studied before.

Evidences indicate that TGFβ-1 signalling is regulated by interleukin-6 (IL-6) activity that promotes transmembrane localization of TGFβ receptors [24]. IL-6 role has been reported in acute phase response, inflammatory cascades, haematopoiesis, hepatic regeneration, metabolism, bone formation, cardiovascular functions, neural development, and innate and adaptive immunity [25]. In HCC, HCV employs IL-6/STAT 3 pathway to cause liver damage [26]. Studies have predicted the association of three IL-6 promoter polymorphisms (rs1800795, rs1800796, rs1800797) with increased risk of cervical cancer, colorectal cancer, breast cancer, prostate cancer, lung cancer, glioma, non-Hodgkin’s lymphoma, and Hodgkin’s lymphoma [27–29]. Studies have demonstrated the association of IL-6 -174G/C with HCC [30]. However, no investigation has been done to study its association with HCV-induced HCC till date.

This study employed an *in-silico* approach to determine pathogenicity of TGFβ-1 and IL-6 gene variants (rs1800470 and rs1800795, respectively) in HCV-induced HCC followed by their validation through allele-specific ARMS-PCR. This approach will aid the earlier diagnosis of HCC by screening the patients against the pathogenic SNPs.

## Materials and methods

### In silico method

#### SNP analysis

IL-6–174 G/C (rs1800795) polymorphism is present in the promoter region, whereas the TGFβ-1 +29C/T polymorphism resides in the coding region. RegulomeDB [31] was used to predict the functional and regulatory role of the variants understudy [32]. The transcription factor binding sites in the promotor region of mutant and wildtype IL-6 genes were found using the AliBaba2 online server [33]. Five algorithms including SIFT, CADD, REVEL, PolyPhen 2.0 and MetaLR were used to assess the potential pathogenicity of the TGFβ-1 human missense variants. The structural differences in the TGFβ-1 variant due to the T^29^→ C mutation were analysed through Project HOPE [34]. Further, TGFβ-1 structure with ID: AF-P01137-F1 was retrieved from Alphafold database [35] and mutation was introduced through wizard tool of PyMol [36]. Interactome analysis was performed through Dynamut software [37].

### In vitro method

#### Study design and patient selection

A retrospective case-control study was conducted in which patients were recruited from the Combined Military Hospital Rawalpindi, Pakistan. The sample size (N) was 429, out of which 213 were HCC patients and 216 were control patients (S1 Data). Patients who developed HCC due to HCV infection were included and all other HCC patients were excluded. Healthy subjects were used as control. Sample size was estimated through the procedure explained by Suresh and Chandra Hekara (2015) and validated using the G*Power Software version 3.1.9.2 for Windows. Sample collection was performed after a written consent of the patient. The study was approved by the Institutional Review Board committee, National University of Sciences and Technology, Islamabad, Pakistan.

### Design of primers

Primer1 was used for designing the allele-specific primer sets for IL-6–174 G/C and TGFβ-1 +29 C/T genes [38]. For IL-6 SNP analysis, the sequence of common primer was 5’-GAGCTTCTCTTTCGTTCC-3’. The sequences for G and C allele primers were 5’-CCCTAGTTGTGTCTTGCG-3’ and 5’-CCCTAGTTGTGTCTTGCC-3’ respectively. For SNP analysis of TGFβ-1, the sequence for common primer was 5’-GTTGTGGGTTTCCACCATTAG-3’ and that for C and T alleles were 5’-CTCCGGGCTGCGGCTGCTGCC-3’ and 5’-CTCCGGGCTGCGGCTGCTGCT-3’, respectively. The sense and antisense internal control primer sequences were 5’-ACACAACTGTGTTCACTAGC-3’ and 5’-CAACTTCATCCACGTTCACC-3’, respectively.

### Genetic analysis

Total DNA was isolated from blood using standardized phenol-chloroform protocol [39]. For the genotyping of IL-6–174 G/C and TGFβ-1 +29C/T polymorphisms, ARMS-PCR was performed using the DreamTaq Green PCR Master Mix (2X). DNA quantity used for genotyping reaction was 10ng/μl. The ARMS-PCR product was analysed through horizontal gel electrophoresis using 2% agarose gel.

### Liver function test

Samples obtained were first screened for infection through ELISA and the samples that were positive for the test were then subjected to ALT, ALP, and AST. The levels of ALT, ALP, and AST in blood serum of patients and healthy individuals were measured through Microlab 300 apparatus (Merck). Comparison of ALT level (IU/L) of patient and control was made to determine the healthy functioning of the liver.

### Statistical analysis

Microsoft office 2016 Excel (Rehmond, WA, USA) was employed to organize the data obtained through experiments. Statistical analysis was performed through the Graph Pad Prism 7 software (GraphPad Software Inc., San Diego, CA, USA). Genotype distribution of studied IL-6 and TGFβ-1 polymorphism and their association strength (odds ratio and relative risk) in HCV-induced HCC patients and healthy control was calculated through two-way Fisher’s exact test. Odds ratio and relative risk was computed through Koopman asymptotic score and Baptista-Pike method, respectively. Odds ratio represents the odds of the disease to occur in an individual with the particular genotype. Relative risk, in contrast, is the probability of the disease occurrence if the individual has the particular genotype. A probability of less than 0.05 was taken as significant.

## Results

### Demographics of the study population

Demographics of study population can be seen in Table 1. Very fewer cases were reported in the 1–20 age group. Greater number of experimental samples fall in the 21–40 & 41–60 age groups. The disease burden progressively decreases in the elderly population with the least number of cases being reported in 61–80 years of age.

**Table 1:** Demographic characteristics of the study groups.

	Patient		Control	
Gender	Male	Female	Male	Female
**Distribution**	91 (42.72%)	122 (57.28%)	103 (47.69%)	113 (52.31%)
**Age in years (Mean ± SD)**	36.91 ± 12.88	34.56 ± 11.70	41.06 ± 13.99	39.11 ± 11.98

SD: Standard Deviation, HCC: Hepatocellular Carcinoma

### Annotation of TGFβ-1 +29 C/T and IL-6–174 G/C variants

RegulomeDB gave annotation score and rank of the TGFβ-1 +29 C/T and IL-6–174 G/C variants, which provided information about the functional impact of these polymorphisms (Table 2). RegulomeDB rank assigned to both mutants was 4, suggesting the presence of transcription factor binding sites and DNase peak [31]. The probability score for both TGFβ-1 +29 C/T and IL-6–174 G/C variants was predicted to be 0.61. If the score is closer to 1, it suggests a regulatory function of the mutant [31]. Therefore, these IL-6 and TGF-β1 variants are predicted to have a regulatory role.

**Table 2:** RegulomeDB analysis of IL-6 (rs1800796) and TGF-β1 (rs1800470) variants.

Variant ID	Functional Rank	Probability Score	Gene Coordinates
IL-6 (rs1800795)	4	0.60906	chr7:22766644–22766645
TGFβ-1 (rs1800470)	4	0.60906	chr19:41858920–41858921

### In silico pathogenicity prediction of TGFβ-1 +29C/T polymorphisms

The pathogenicity scores and deleteriousness of TGFβ-1 +29C/T (rs1800470) polymorphism as predicted by SIFT, CADD, REVEL, PolyPhen 2.0, and MetaLR algorithms can be viewed in Table 3. All five tools have predicted the clinical significance of this mutant as benign.

**Table 3:** TGF-β1 +29C/T variant as predicted by SIFT, CADD, REVEL, PolyPhen and MetaLR tools.

Tool	Reference Value	Qualitative prediction	Obtained Score	Inference
SIFT	< 0.05	Deleterious	0.51	Tolerated
	≥ 0.05	Tolerated		
CADD	> 30	Likely deleterious	18	Likely benign
	< 30	Likely benign		
REVEL	> 0.5	Likely disease causing	0.03	Likely benign
	< 0.5	Likely benign		
Meta-LR	Score between 0 & 1	Either are tolerated or damaging	0.034	Tolerated
PolyPhen	> 0.908	Probably damaging	0	Benign

**Fig 1:**
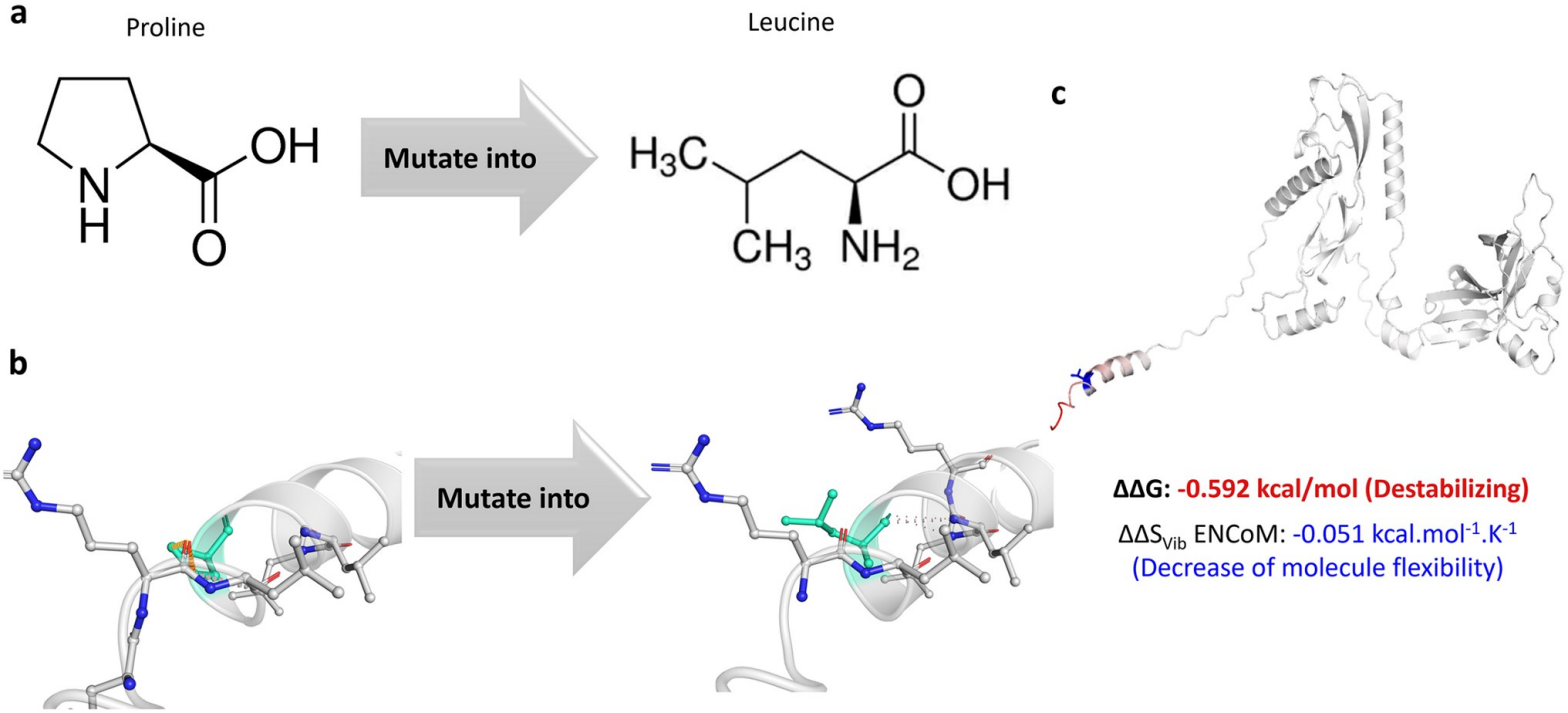
Proline to leucine amino acid substitution in TGFβ-1. **a)** The structure of proline (left) and leucine (right). The backbone structure, coloured red, remains the same for both. Whereas the side chain (coloured black) is unique for each amino acid. **b)** Missense mutation impact on the inter-molecular interactions of TGFβ-1. Green coloured amino acid represent wildtype and altered amino acids. **c)** Molecular flexibility analysis of TGFβ-1.

The C/T polymorphism of the TGFβ-1 protein leads to an amino acid substitution of a proline to a leucine at position 10 (Fig 1a). According to project HOPE, the wildtype and mutant amino acids differ in sizes. The mutant residue is larger in size, which may introduce bumps in the protein structure. As proline is known to have a rigid structure which is essential for maintaining the protein backbone, its mutation may have caused changes in the structural confirmation of the TGFβ-1 protein. Interactome interaction analysis revealed that mutation caused the loss of ionic bonds and gain of hydrophobic interactions and hydrogen bonds in the protein (Fig 1b). Furthermore, P10L variation decreases molecular flexibility and is destabilizing in nature (Fig 1c).

### In silico pathogenicity prediction of IL-6–174 G/C polymorphisms

Alibaba2 predicted five transcription factor binding sites for the wildtype IL-6 gene. These included CREB, CRE-BP1, CPE_bind, c-FOS, and C/EBPbeta sites. However, an additional binding site of nuclear factor-1 (NF-1) was predicted in the mutant IL-6 sequence along with the wildtype transcription factor binding sites. Table 4 encloses the binding sites and their nucleotide ranges for both mutant and wild type IL-6 gene.

**Table 4:** AliBaba2 results tabulated to represent segments for the transcription factor binding sites and their nucleotide ranges in mutant IL-6 gene.

Segments	Nucleotide Range	Binding Sites
9. 9. 539	9–18	NF-1
1. 1. 2. 0	24–33	CREB
1. 1. 1. 6	25–34	CRE-BP1
2. 3. 3. 0	25–34	CPE_bind
1. 1. 1. 2	29–38	c-Fos
1. 1. 3. 0	33–44	C/EBP beta

NF-1: Nuclear Factor-1, CREB: cAMP response element-binding protein

### TGFβ-1 +29 C/T and IL-6–174 G/C polymorphic distribution amongst study groups

Out of all three genotypes of TGFβ-1+29 C/T, TT is the most prevalent (24.94%) and presents a positive association with the development of HCV-induced HCC (p<0.0001, OR = 5.403, RR = 2.062). The CT and CC genotypes have a relatively greater distribution in the control group (26.81% and 15.62%, respectively) as compared to the HCC group (15.85% and 8.86%, respectively). A negative correlation existed between cancer development and CT (p<0.0001, OR = 0.4119, RR = 0.6304) and CC (p = 0.001, OR = 0.4829, RR = 0.67) genotypes, respectively (Table 5).

**Table 5:** Individual representation of TGF-β1 +29C/T and IL-6 -174G/C genotypes amongst HCC and control groups.

Genotypes	Distribution		Odds Ratio	95% CI-Odds Ratio	Relative Risk	95% CI-Relative Risk	p-value
	Patient	Control					
*TGF-β1*							
TT	24.94%	7.93%	5.40	3.42 to 8.56	2.06	1.72 to 2.46	<0.0001
CT	15.85%	26.81%	0.41	0.27 to 0.61	0.63	0.50 to 0.77	<0.0001
CC	8.86%	15.62%	0.48	0.30 to 0.75	0.67	0.50 to 0.86	0.001
*IL-6*							
GG	9.09%	21.68%	0.29	0.19 to 0.45	0.51	0.37 to 0.65	<0.0001
GC	29.93%	19.72%	2.31	1.57 to 3.36	1.52	1.25 to 1.85	<0.0001
CC	10.47%	8.84%	1.24	0.77 to 2.04	1.11	0.87 to 1.37	0.39

Amongst the IL-6–174 G/C genotypes, GC has the maximum frequency distribution in the HCC group (29.6%). Moreover, GC genotype carriers have a positive correlation with HCC development (p<0.0001, OR = 2.276, RR = 1.512). Subjects with GG genotype present a lower distribution in the HCC group (8.39%) and a negative association with cancer development (p<0.0001, OR = 0.2456, RR = 0.4685). No statistically significant difference was observed in the susceptibility of the disease amongst CC genotype carriers (p = 0.39). The genotypic distribution of TGFβ-1 and IL-6genotypes is enclosed in Table 5.

### Association of TGFβ-1 +29 C/T and IL-6–174 G/C genotypes with gender in HCV-induced HCC and controls

In the gender dependent distribution of TGFβ-1 +29 C/T genotypes, TT is the predominant genotype in males (29.77%) and females (20.09%). The results suggest a strong association of the TT genotype with the development of HCC in both males (p<0.0001, OR = 6.531, RR = 2.161) and females (p<0.0001, OR = 4.304, RR = 1.936). The CT genotype was found to be poorly distributed and negatively associated with the risk of developing cancer in patients (male: p = 0.0004, 13.02%; female: p = 0.0141, 18.69%). Similarly, only 9.77% men with HCC had CC genotype (p = 0.01) and presented a negative association with HCC susceptibility. However, no statistically significant difference has been observed in the association of liver cancer development with the CC genotype in the female gender (p = 0.07) (Table 6).

**Table 6:** Association of TGFβ-1 +29 C/T and IL-6–174 G/C genotypes with gender in HCV-induced HCC and control patients.

Genotypes	HCV-induced HCC Patients (%)	Control (%)	Odds Ratio	95% CI-Odds Ratio	Relative Risk	95% CI-Relative Risk	p-value
*TGF-β1*							
TT-M	29.77%	7.91%	6.53	3.44 to 12.53	2.16	1.69 to 2.79	<0.0001
CT-M	13.02%	23.26%	0.34	0.19 to 0.61	0.57	0.41 to 0.78	0.0004
CC-M	9.77%	16.28%	0.43	0.23 to 0.83	0.64	0.44 to 0.90	0.01
TT-F	20.09%	7.94%	4.30	2.21 to 8.38	1.93	1.48 to 2.50	<0.0001
CT-F	18.69%	30.37%	0.50	0.28 to 0.85	0.69	0.51 to 0.92	0.01
CC-F	7.94%	14.95%	0.52	0.26 to1.01	0.68	0.44 to 1.01	0.07
*IL-6*							
GG-M	8.29%	27.46%	0.19	0.10 to 0.39	0.38	0.24 to 0.58	<0.0001
GC-M	27.46%	16.58%	2.91	1.71 to 5.36	1.77	1.31 to 2.35	0.0003
CC-M	11.4%	8.81%	1.59	0.78 to 3.15	1.25	0.87 to1.69	0.21
GG-F	9.75%	16.95%	0.42	0.24 to 0.76	0.63	0.44 to 0.88	0.005
GC-F	32.20%	22.46%	1.90	1.13 to 3.13	1.37	1.06 to 1.79	0.01
CC-F	9.75%	8.90%	1.029	0.52 to 1.93	1.014	0.71 to1.34	>0.9999

CI: Confidence Interval, HCC: Hepatocellular Carcinoma, M: Male, F: Female

The distribution of IL-6–174 G/C genotypes represents that GC is the most prevalent genotype amongst male and female HCV-induced hepatic cancer patients (male = 27.46%, female = 32.20%). The data suggests that there is a high risk of developing HCC in male (p = 0.0003, OR = 2.917, RR = 1.772) and female (p = 0.01, OR = 1.902, RR = 1.370) carriers of GC genotype. Contrary to GC, the distribution of the GG genotype was lower in HCC patients as compared to their healthy counterparts in both male (8.29% vs 27.46%) and female (9.75% vs 16.95%) genders, respectively. The analysis of GG genotype indicates its protective role against liver carcinogenesis in both men (p<0.0001, OR = 0.1972, RR = 0.3834) and women (p = 0.005, OR = 0.4298, RR = 0.6380). The gender distribution of the IL-6 genotypes can be seen in Table 6.

### Co-occurrence of TGFβ-1 +29 C/T and IL-6–174 G/C polymorphic distribution amongst HCV-induced HCC and control

In current study, influence of the co-existence of IL-6 and TGFβ-1 genotypes in HCV-mediated HCC patients is also investigated. Co-occurrence of genotypes GC (IL-6) and TT (TGFβ-1) was found in 26.85% of patients and has significant association with HCV-mediated HCC (p<0.0001). Further, OR and RR indicated that their co-existence enhances the probability of HCC development in HCV patients. Likewise, co-existence of genotypes GG (IL-6) and CC (TGFβ-1) and genotypes GG (IL-6) and TC (TGFβ-1) was in 3.24% and 3.70% patients, respectively and has been identified as protective (Table 7).

**Table 7:** TGFβ-1 and IL-6 co-existing genotypes, along with their relative risk, odds ratio and p-value in HCV-induced HCC patients.

Genotype (IL6+ TGFβ-1)	Number		Relative Risk	95% CI	Odds Ratio	95% CI	P-value
	Patients (%)	Control (%)					
GG+TT	24 (11.11)	15 (7.08)	1.24	0.91 to 1.56	1.64	0.82 to 1.56	0.1793
GG+TC	8 (3.70)	48 (22.64)	0.25	0.13 to 0.46	0.13	0.06 to 0.27	<0.0001
GG+CC	7(3.24)	29(13.68)	0.36	0.18 to 0.66	0.21	0.08 to 0.49	<0.0001
GC+TT	58(26.85)	12(5.66)	1.87	1.58 to 2.18	6.11	3.14 to 11.76	<0.0001
GC+TC	47(21.76)	44(20.75)	1.03	0.81 to 1.27	1.06	0.67 to 1.67	0.8142
GC+CC	26(12.04)	27(12.74)	0.96	0.70 to 1.25	0.93	0.52 to 1.65	0.8838
CC+TT	27(12.50)	6(2.83)	1.71	1.34 to 2.01	4.91	2.07 to 11.43	0.0002
CC+TC	13(6.02)	23(10.85)	0.69	0.43 to 1.02	0.52	0.25 to 1.04	0.0824
CC+CC	6(2.78)	8(3.77)	0.72	0.24 to 2.16	0.84	0.41 to 1.34	0.5982

### Comparison of ALT, ALP, and AST of HCV-induced HCC with control

**Fig 2:**
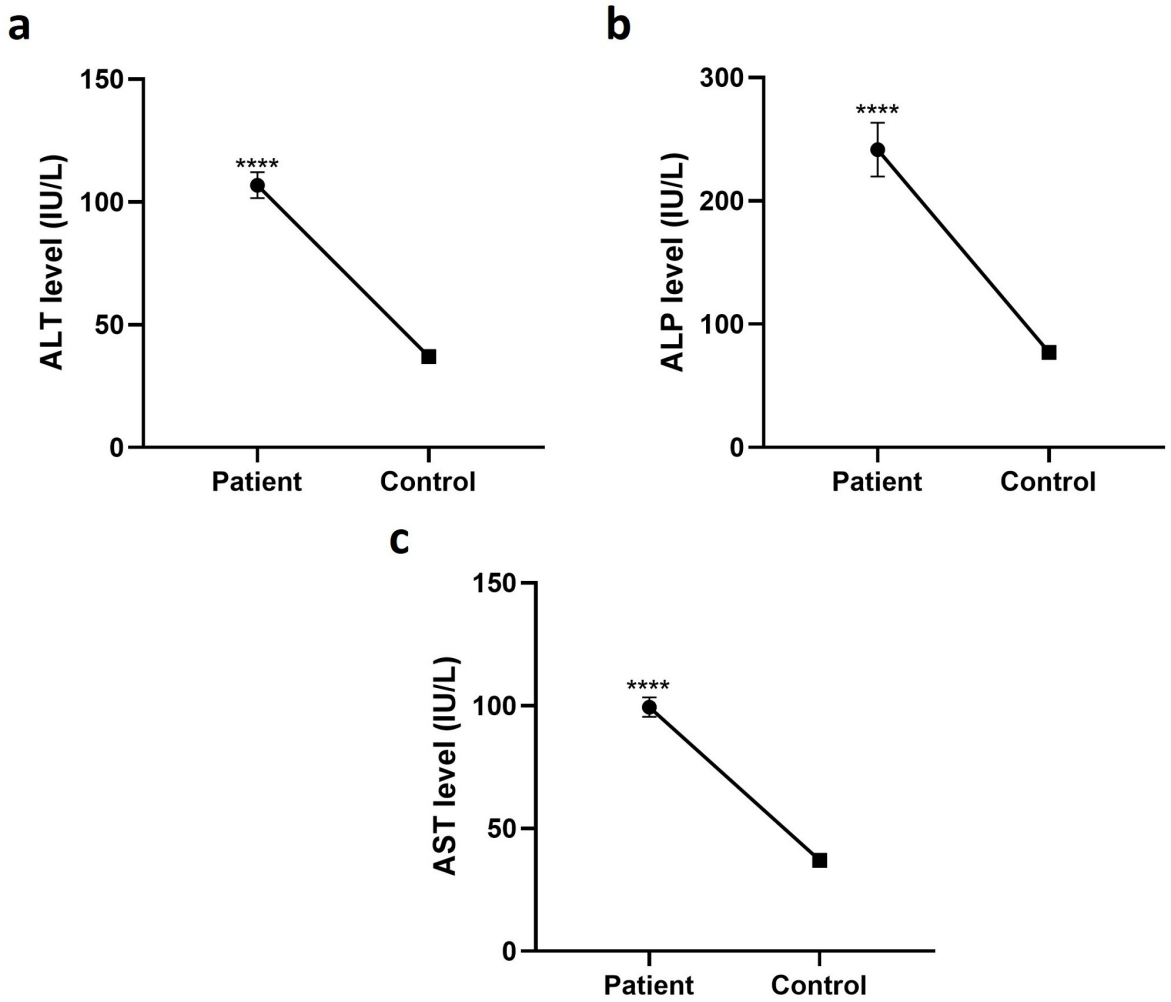
Comparison of ALT, ASP, and ALP levels in HCV-induced HCC patients and healthy individuals. Mean ALT, ALP, and AST levels in patients were higher than control. Data is represented as mean ± SD. **** represents p<0.0001. P-value below 0.05 is considered significant.

ALT, AST, and ALP of HCV-mediated HCC patients and healthy control was compared through independent *t*-test. Analysis revealed that ALT, AST, and ALP level in HCV-induced HCC patients was elevated relative to control with P value <0.0001. Mean ALT level found in patients was 107 with standard deviation of 52, ALP mean ± SD 241 ± 16, and AST mean ± SD 99 ± 40. Similarly, the value of ALT in control was mean ± SD 37 ± 10, ALP mean ± SD 77 ± 16, and AST mean ± SD 37 ± 13. Fig 2 illustrates the mean ALT, AST, and ALP level in HCV-induced HCC patients and healthy individuals.

## Discussion

HCC is marked as the most common liver malignancy, especially in HBV and HCV-infected patients. The life expectancy of HCC depends on the progression of disease and stage of cancer. When diagnosed at an early stage, the treatment may be effective. However, at an advanced stage the therapy does not produce effective results [40]. Multiple studies have reported an association of IL-6–174 G/C polymorphism with the pathogenesis of liver diseases including HCC derived from HCV [41, 42]. To our knowledge, no studies on the association of TGFβ-1 +29 C/T polymorphism with HCV-induced HCC are available. This study aims to assess the risk of developing hepatic cancer in HCV-infected patients due to the presence of IL-6–174 G/C and TGFβ-1 +29 C/T polymorphisms.

Despite the reported deleterious nature of this variant, the bioinformatics analysis classified the clinical significance of TGFβ-1 +29C/T polymorphism as benign. In silico bioinformatics tools employed for SNP analysis usually determine genetic variant pathogenicity based on evolutionary conservation and sequence homology [43]. Classification of TGFβ-1 +29C/T polymorphism as neutral or benign by SIFT, PolyPhen2.0, REVEL, CADD and MetaLR indicates the need to update the algorithm of these tools, so more accurate predictions relevant to SNP pathogenicity can be made. These tools use sequence information and employ evolutionary conservation status, homology sequences, amino acids’ physical properties, structural information, and human protein’s function to make prediction. These tools must include information from experimental data to make the outcomes of the tools more accurate. Further, their algorithm must be updated from time to time to give more precise results. According to RegulomeDB analysis, both the IL-6 -174G/C and TGFβ-1 +29C/T polymorphisms are predicted to have regulatory roles as their probability scores (0.60906) were closer to 1.

An additional nuclear factor-1 (NF-1) binding site, based on 9 nucleotides, was identified in the IL-6 variant as compared to the wildtype sequence. NF-1, also known as the CAAT box-binding transcription factor (CTF), is a wide family of DNA-binding proteins that recognize and bind to the CAAT box (GCCAAT sequence) in the promotor region. The CTF/NF-1 proteins regulate the gene expression by acting as transcription factors for RNA polymerase II. Moreover, the CTF/NF-1 proteins also function as initiation factors for recruiting DNA polymerase for adenovirus DNA replication [20, 44]. The binding sites for CTF/NF1 have been reported in the promotor, enhancer, and silencer regions of a gene. The alternative splicing generates multiple variants of NF-1 protein that function as activators or repressors of transcription [45]. Nuclear factor is previously been demonstrated to have role in IL-6 expression [46]. Some studies have formed an association of increased IL-6 serum levels with the presence of IL-6 -174G/C polymorphism [47, 48]. IL-6 enhanced serum concentration has been reported to have association with HBV-induced HCC [49]. Similarly, IL-6 enhanced level in HCV also provides advantage to cancer cells by suppressing apoptosis, hence leading to hepatic cancer development [50]. So, results from AliBaba suggests the introduction of new transcription binding site due to IL-6 polymorphism might play part in up-regulating its expression in HCV-induced HCC.

Evidence from literature suggests that approximately 15% of the human codons have dual function. Such codons can simultaneously code of particular amino acids and specify transcription factor recognition sites and may refer to as “duons” [51]. RegulomeDB estimated regulatory function of TGFβ-1 polymorphism that is in protein-coding region. No study is found so far that suggest the binding of transcription factor to exonic region of TGFβ-1. However, duon mutations have been identified in TPD53 and SF3B1 that also has correlation with cancer development and progression [52]. These outcomes suggest the commencement of further deep analysis to delineate the impact of SNPs on the functional as well as regulatory role of proteins.

Structural analysis revealed that TGFβ-1 polymorphism induces wildtype proline substitution with mutant leucine at amino acid residue 10. Proline has a side chain that is covalently bonded to the peptide backbone to form a cyclized structure [53, 54]. This cyclic structure confers rigidity to the protein [55, 56]. Proline inhibits the secondary and tertiary confirmation of protein because it does not allow the formation of alpha-helix or beta-sheet structure. Instead, it introduces breaks and links into the alpha-helical part of the protein backbone [53, 54]. On the other hand, leucine has a hydrophobic aliphatic side chain which favours the formation of alpha-helical structure [54, 57]. This suggests that the Pro10Leu mutation may have caused changes in the binding pattern and rigidity of the TGFβ-1 variant as compared to its wildtype counterpart. Moreover, leucine is larger in size than proline. The Pro10Leu mutation may have caused the formation of bumps in mutant TGFβ-1 protein structure, as predicted by Project HOPE.

The Pro10Leu polymorphism lies in the signal peptide domain of the TGF-β1 protein. A signal peptide directs a newly synthesised protein to the ER for further processing [58]. Based on the chemical differences in proline and leucine, a study has previously hypothesized that the Pro10Leu mutation may influence the binding of the nascent protein to the TGFB1 protein, thus impacting the peptide export efficiency of the TGF-β1 [59]. This may influence the availability of the nascent protein. However, more studies are required to validate this.

Studies performed on breast cancer, and colorectal cancer identified a positive association of IL-6–174 G/C polymorphism with HCC development [60, 61]. A meta-analysis study has also confirmed a significant association of IL-6–174 G/C polymorphism with HCC susceptibility [62]. Some groups have studied the association of SNPs in IL-6 gene with the development of HCC in Pakistani population [26, 63], however, none of these studies focused on the -174G/C polymorphism in HCV induced HCC. Badshah et al. studied IL-6-174G/C polymorphisms in association with the pathogenesis and prognosis of HCV infection. The study reported a high prevalence of homozygous genotypes (GG and CC) but the heterozygous GC genotype was identified to be protective against HCV infection [63]. On the other hand, the results of our study suggest that the GC genotype holds a positive association, and the GG genotype holds a negative association against the development of HCC. Comparison of both studies’ outcome suggests that IL-6 genotype GC has protective role in HCV but enhances risk of HCC development in HCV patients. It creates room for further transcriptome and proteome level investigations for better understanding the role of IL-6 -174G/C polymorphisms in HCV infection in both HCC and non-HCC cases to strengthen the validity of reported results.

A study on the colon cancer patients has identified the negative association of GG genotype of TGFβ-1 +29 C/T with cancer which is suggestive of its protective impact against the disease [64]. The GC genotype was found to have a higher prevalence in male patients as compared to female patients, which could be a possible explanation of the higher disease burden of HCC in men.

A research performed on bladder cancer patients in Indian population has reported the TT homozygous genotype to be disease-causing [19]. Another group studying TGFβ-1 +29 C/T polymorphism in breast cancer patients in India also identified TT to be a pathogenic genotype [65]. This is consistent with our finding of TT genotype having a pathogenic association with HCC development in both males and females in Pakistani population. No data has been reported previously on the possible association of CC or CT genotypes with HCC pathogenesis. T allele has been reported to be a disease associated allele whereas no such association has been established for the C allele [65]. This suggests that the CC genotype might hold a protective role against HCC development. This is in accordance with our findings of poor association of CC genotype with cancer prevalence. Another research on investigating the role of TGFβ-1 +29 C/T in prostate cancer also found no significant association of CC genotype with the cancer development [66].

It is delineated in current study that co-occurrence of TGFβ-1 TT genotype and IL-6 GC genotype in HCV patient increases the risk of HCC development. Previously, the association of these two polymorphisms with recurrent spontaneous abortions in Brazilian women is investigated but no conclusive outcomes regarding their co-occurrence were drawn [67]. However, the co-existence of these two genotypes has been reported to cause hip dysplasia in osteoarthritis patients [18].

## Conclusion

TGFβ-1 +29C/T and IL-6 -174G/C polymorphisms are involved in the carcinogenesis of HCC in HCV-infected population. TT genotype of TGFβ-1 gene and the GC genotype of IL-6 gene are found to be deleterious due to their contribution towards the development of HCC in patients. The co-occurrence of TGFβ-1 TT genotype and IL-6 GC genotype in HCV infected individual poses high risk of HCC development. These findings propose that an early diagnosis of HCC may be possible through genotyping of HCV patients.

## Supporting information

S1 Data Genotyping data of control and patient group for IL-6 and TGFβ-1 gene. (XLSX)
